# A Lumped Two-Compartment Model for Simulation of Ventricular Pump and Tissue Mechanics in Ischemic Heart Disease

**DOI:** 10.3389/fphys.2022.782592

**Published:** 2022-05-11

**Authors:** Tijmen Koopsen, Nick Van Osta, Tim Van Loon, Frans A. Van Nieuwenhoven, Frits W. Prinzen, Bas R. Van Klarenbosch, Feddo P. Kirkels, Arco J. Teske, Kevin Vernooy, Tammo Delhaas, Joost Lumens

**Affiliations:** ^1^ Department of Biomedical Engineering, Cardiovascular Research Institute Maastricht (CARIM), Maastricht University, Maastricht, Netherlands; ^2^ Department of Physiology, Cardiovascular Research Institute Maastricht (CARIM), Maastricht University, Maastricht, Netherlands; ^3^ Division of Heart and Lungs, Department of Cardiology, University Medical Center Utrecht, Utrecht, Netherlands; ^4^ Department of Cardiology, Cardiovascular Research Institute Maastricht (CARIM), Maastricht University Medical Center, Maastricht, Netherlands; ^5^ Department of Cardiology, Radboud University Medical Center, Nijmegen, Netherlands

**Keywords:** myocardial infarction, computational modeling and simulation, deformation imaging, contractile dysfunction, strain

## Abstract

**Introduction:** Computational modeling of cardiac mechanics and hemodynamics in ischemic heart disease (IHD) is important for a better understanding of the complex relations between ischemia-induced heterogeneity of myocardial tissue properties, regional tissue mechanics, and hemodynamic pump function. We validated and applied a lumped two-compartment modeling approach for IHD integrated into the CircAdapt model of the human heart and circulation.

**Methods:** Ischemic contractile dysfunction was simulated by subdividing a left ventricular (LV) wall segment into a hypothetical contractile and noncontractile compartment, and dysfunction severity was determined by the noncontractile volume fraction ( NCVF ). Myocardial stiffness was determined by the zero-passive stress length ( $L_{s0,pas})$ and nonlinearity ( $k_{ECM}$ ) of the passive stress-sarcomere length relation of the noncontractile compartment. Simulated end-systolic pressure volume relations (ESPVRs) for 20% acute ischemia were qualitatively compared between a two- and one-compartment simulation, and parameters of the two-compartment model were tuned to previously published canine data of regional myocardial deformation during acute and prolonged ischemia and reperfusion. In six patients with myocardial infarction (MI), the NCVF was automatically estimated using the echocardiographic LV strain and volume measurements obtained acutely and 6 months after MI. Estimated segmental NCVF values at the baseline and 6-month follow-up were compared with percentage late gadolinium enhancement (LGE) at 6-month follow-up.

**Results:** Simulation of 20% of NCVF shifted the ESPVR rightward while moderately reducing the slope, while a one-compartment simulation caused a leftward shift with severe reduction in the slope. Through tuning of the NCVF , $L_{s0,pas}$ , and $k_{ECM}$ , it was found that manipulation of the NCVF alone reproduced the deformation during acute ischemia and reperfusion, while additional manipulations of $L_{s0,pas}$ and $k_{ECM}$ were required to reproduce deformation during prolonged ischemia and reperfusion. Out of all segments with LGE>25% at the follow-up, the majority (68%) had higher estimated NCVF at the baseline than at the follow-up. Furthermore, the baseline NCVF correlated better with percentage LGE than NCVF did at the follow-up.

**Conclusion:** We successfully used a two-compartment model for simulation of the ventricular pump and tissue mechanics in IHD. Patient-specific optimizations using regional myocardial deformation estimated the NCVF in a small cohort of MI patients in the acute and chronic phase after MI, while estimated NCVF values closely approximated the extent of the myocardial scar at the follow-up. In future studies, this approach can facilitate deformation imaging–based estimation of myocardial tissue properties in patients with cardiovascular diseases.

## 1 Introduction

Computational modeling of cardiac mechanics and hemodynamics in ischemic heart disease (IHD) is important for better understanding of the complex relations between ischemia-induced heterogeneity of myocardial tissue properties, regional tissue mechanics, and hemodynamic pump function. Spatially detailed three-dimensional models based on the finite element method (FEM) are most frequently used for simulation of myocardial infarction (MI) and its effects on cardiac geometry, tissue mechanics, and electrophysiology. While many studies have successfully used FEM models for investigating the pathophysiology and potential treatment of MI (Fomovsky et al., 2011; Fomovsky et al., 2012; Rouillard and Holmes, 2014; Veress et al., 2015; Leong et al., 2017; Haddad and Samani, 2018; Wang et al., 2018; Chan et al., 2019; Fan et al., 2019; Zhuan et al., 2019; Estrada et al., 2020; Zhang et al., 2020; Zhang et al., 2021), complexity of these models can provide a problem when performing simulations on a patient-specific level (Moulton et al., 2017).

Reduced-order modeling approaches with sufficient spatial and physiological details to accurately simulate global and regional tissue mechanics are important for patient-specific simulation of MI (Moulton et al., 2017; Holmes and Lumens, 2018). The CircAdapt lumped-parameter model of the human heart and circulation is a closed-loop model which simulates real-time, beat-to-beat hemodynamics and mechanics of the heart and blood vessels (Arts et al., 2004; Lumens et al., 2009). Previous studies using CircAdapt have shown that the model realistically simulates global ventricular hemodynamics and regional myocardial mechanics in various pathological conditions (Lumens et al., 2009; Walmsley et al., 2015). However, its ability to simulate the effects of ischemia-induced contractile dysfunction on both global pump and regional tissue mechanics has not been evaluated yet.

In this study, we presented and tested a modeling approach for MI-induced myocardial contractile dysfunction, which is integrated into the CircAdapt modeling framework. Following previous observations by Sunagawa et al. (1982), who showed that global ventricular mechanics during acute regional ischemia were best described using a two-compartment modeling approach, we subdivided an ischemic wall segment into an active and a passive compartment. We validated this two-compartment implementation by comparing its simulated effects of regional ischemia on the global LV pump function against the results of a one-compartment implementation and by tuning parameters of the two-compartment model to mimic existing data on regional myocardial deformation in dogs with coronary artery ligation. We then evaluated whether the model could be used to estimate the severity of regional contractile dysfunction in MI patients by applying an automatic optimization algorithm to measurements of regional myocardial deformation performed within 72 h and after 6 months following MI and by comparing estimated severities to percentage late gadolinium enhancement (LGE) after 6 months.

## 2 Materials and Methods

A brief description of the CircAdapt model components, which are most relevant for this study, are provided as follows. For further details on the CircAdapt model, we referred to previously published validation studies (Walmsley et al., 2015; Lumens et al., 2009). Thereafter, the one- and two-compartment models for ischemia-induced contractile dysfunction were described, and we explained how simulated global mechanics were compared between both modeling approaches. Then, we described how model simulations using the two-compartment model were tuned using existing experimental measurements. Finally, we introduced the clinical data and computational methods used for imaging-based estimation of regional contractile dysfunction in patient hearts acutely and 6 months after MI.

### 2.1 CircAdapt Model

The CircAdapt computational model of the human heart and circulation is a closed-loop lumped-parameter model that simulates beat-to-beat hemodynamics and mechanics of the heart and blood vessels (Arts et al., 2004; Lumens et al., 2009). The model uses a simplified ventricular geometry, where cardiac walls are represented by thick-walled spherical shells consisting of myofibers. The TriSeg module allows for interventricular interaction by coupling the left (LV) and right ventricular (RV) walls through the interventricular septum (Lumens et al., 2009). Walls can be subdivided into patches using the MultiPatch module (Walmsley et al., 2015), which enables heterogeneity of myocardial tissue properties within the walls. Myofiber active and passive stress generation is modeled using a three-element Hill model (Arts et al., 2003). In brief, the series contractile element with the time-dependent length $L_{si}\left(t\right)$ generates an active stress $\sigma_{f,act}\left(t\right)$ which represents the force developed through cross-bridge formation between actin and myosin filaments. The zero-active stress contractile element length $L_{si0,act}$ defines the length at which no active stress is developed. The series elastic element with the time-dependent length $L_{se}\left(t\right)$ describes the intrinsic elasticity of the sarcomere, and a reference length of $L_{se,iso}$ is used to define the length of the series elastic element at the onset of isovolumetric contraction. Furthermore, the density of cross-bridge formation is described by a time-dependent contractility state variable $C\left(t,\;L_{si}\left(t\right)\right)$ , while active stress generation is scaled by a parameter $S_{f,act}$ . Taken together, active stress is calculated as follows:

$$\sigma_{f,act}\left(t\right)=\;S_{f,act}.C\left(t,\;L_{si}\left(t\right)\right).\left(L_{si}\left(t\right)-L_{si,act}\right).\frac{L_{se}\left(t\right)}{L_{se,iso}}$$ (1)

Passive myocardial tissue behavior is described by the parallel elastic element, which captures both extracellular and intracellular structures. Therefore, total passive stress $\sigma_{f,pas}\left(t\right)$ is the sum of the extracellular matrix stress $\sigma_{f,ECM}\left(t\right)$ and intracellular stress, that is, titin stress $\sigma_{f,tit}\left(t\right)$ :

$$\sigma_{f,pas}\left(t\right)=\;\sigma_{f,ECM}\left(t\right)+\;\sigma_{f,tit}\left(t\right)$$ (2)

The extracellular matrix stress $\sigma_{f,pas}\left(t\right)$ depends as follows on the total time-dependent sarcomere length $L_{s}\left(t\right)$ , which is the sum of $L_{si}\left(t\right)$ and $L_{se}\left(t\right)$ :

$$\sigma_{f,ECM}\left(t\right)=\;S_{f,pas}.\left({\left(\frac{L_{s}\left(t\right)}{L_{s0,pas}}\right)}^{k_{ECM}}-1\right)$$ (3)

Here, $S_{f,pas}$ is a parameter scaling passive stress development, while $L_{s0,pas}$ is the zero-passive stress sarcomere length. The parameter $k_{ECM}$ determines the nonlinearity of the relation. The titin stress $\sigma_{f,tit}\left(t\right)$ depends on $L_{s}\left(t\right)$ , $S_{f,act}$ , $L_{s0,pas}$ , and two parameters which scale the development of titin stress ( $k_{1,tit}$ ) and the nonlinearity of the relation ( $k_{2,tit}$ ):

$$\sigma_{f,tit}\left(t\right)=\;S_{f,act}.k_{1,tit}.\left({\left(\frac{L_{s}\left(t\right)}{L_{s0,pas}}\right)}^{k_{2,tit}}-1\right)$$ (4)

### 2.2 Simulating Ischemia-Induced Contractile Dysfunction: One-Compartment Vs. Two-Compartment Model

**FIGURE 1:**
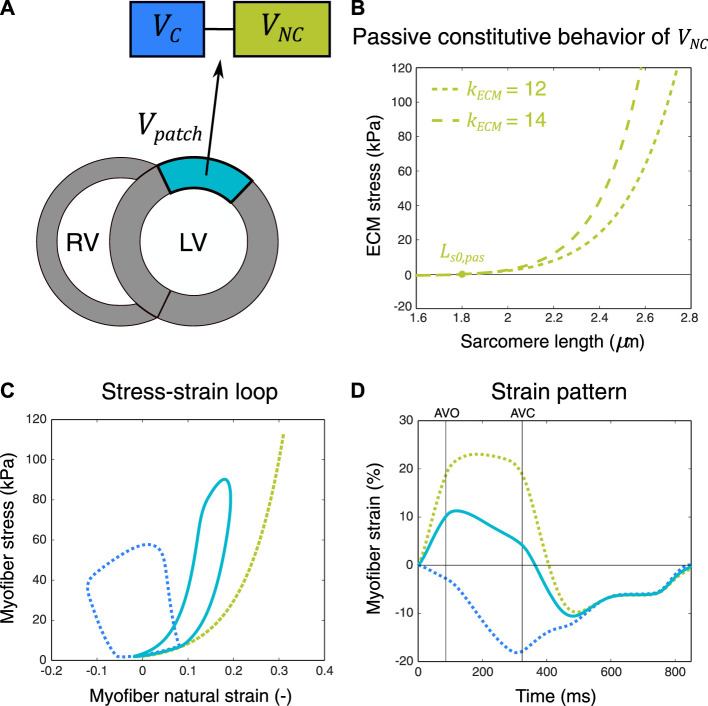
Schematic representation of the two-compartment modeling approach used for simulation of ischemic myocardial dysfunction. A myocardial patch with volume $V_{patch}$ was subdivided into a hypothetical contractile and noncontractile patch (i.e., compartment) with volumes $V_{C}$ and $V_{NC}$ , respectively, being serially coupled **(A)**. Myocardial volume $V_{C}$ generated active stress, while $V_{NC}$ was passive **(C)**. Myocardial stiffness was determined by passive constitutive behavior of $V_{NC}$ , determined by the zero-passive stress sarcomere length ( $L_{s0,pas}$ ) and nonlinearity parameter ( $k_{ECM}$ ) **(B)**. The resulting strain pattern of $V_{patch}$ **(D)** and its stress–strain loop **(C)** were calculated from the volume-weighted average sarcomere length and stress of $V_{C}$ and $V_{NC}$ . AVO, aortic valve opening; AVC, aortic valve closure.

In a combined experimental and modeling study, Sunagawa et al. (1982) showed that the LV systolic function during regional ischemia was best captured by a two-compartment model including an active and a passive compartment. The rationale behind this two-compartment model is that the active compartment lumps all of the normally functioning tissues within the nonischemic and ischemic region, while the passive compartment lumps all of the nonfunctional parts of the ischemic region. As mentioned before, we hypothesized that we could use a similar modeling approach not only for simulating global LV pump mechanics during regional ischemia but also to simulate average regional LV tissue mechanics (i.e., the myofiber stress and strain) in ischemic myocardial segments, which are defined by deformation imaging techniques, for example, speckle tracking echocardiography (STE). In the CircAdapt model, tissue properties are defined per myocardial patch, and by default, each myocardial wall includes one myocardial patch. The active and passive stress of a patch is calculated using Eqs 1–4. To simulate reduced patch contractility, one approach is to reduce parameter $S_{f,act}$ (Eq. 1). Since this approach does not involve subdivision of the myocardial patch into multiple patches (i.e., compartments), we term this as a one-compartment modeling approach for simulating contractile dysfunction. For the two-compartment model of ischemia, the CircAdapt MultiPatch module (Walmsley et al., 2015) is applied. The MultiPatch module has previously been used to subdivide myocardial walls into different patches, each having their own unique tissue properties (Walmsley et al., 2015; Lumens et al., 2015; Voigt et al., 2015). In this study, we applied the MultiPatch module to further subdivide an ischemic myocardial patch with volume $V_{patch}$ into a contractile and a noncontractile patch (i.e., compartment) with volumes $V_{C}$ and $V_{NC}$ , respectively (Figure 1). Compared to the implementation of Sunagawa et al. (1982), where the subdivided volume $V_{patch}$ equaled the volume of the entire LV wall, we additionally applied the two-compartment model on a regional level, where $V_{patch}$ equals the volume of an echocardiographic segment by approximation. Mechanics of $V_{C}$ are described by Eqs 1–4, while for $V_{NC}$ it holds $S_{f,act}=0$ ; therefore, the mechanics of $V_{NC}$ are fully described by Eq. 3. The degree of patch contractile dysfunction is determined by the noncontractile volume fraction ( NCVF ), defined as the relative volume of the noncontractile compartment:

$$NCVF=\;\frac{V_{NC}}{V_{patch}}$$ (5)

### 2 3.Myofiber Strain Calculations

The calculated strain ε(t) during the cardiac cycle represented the engineering strain based on the sarcomere length $L_{s}\left(t\right)$ :

$$\varepsilon \left(t\right)=\left(\frac{L_{s}\left(t\right)}{L_{s,ref}}-1\right).100\%$$ (6)

where $L_{s,ref}$ is a reference sarcomere length used for strain calculations.

For the two-compartment model, the sarcomere length $L_{s}\left(t\right)$ of $V_{patch}$ was calculated as the volume-weighted average of the sarcomere lengths of the contractile and noncontractile compartments, respectively, $L_{s,C}\left(t\right)$ and $L_{s,NC}\left(t\right)$ :

$$L_{s}\left(t\right)=\left(1-NCVF\right).L_{s,C}\left(t\right)+\;NCVF.L_{s,NC}\left(t\right)$$ (7)

### 2.4 Simulation Protocol

#### 2.4.1 Global Myocardial Mechanics: One-Compartment Vs. Two-Compartment Model

In line with the study of Sunagawa et al. (1982), we compared simulated LV pump mechanics during acute regional ischemia when using a two-compartment model against a one-compartment model by constructing end-systolic pressure volume relations (ESPVRs). We started with a baseline reference simulation with a heart rate of 71 bpm, stroke volume (SV) of 72 ml, and a mean arterial pressure (MAP) of 92 mmHg. Homeostatic pressure-flow regulation was enabled, meaning that MAP and SV were kept constant through regulation of the peripheral arterial resistance and circulating blood volume. First, for the two-compartment simulation, the NCVF was set to 0.2 in the LV free wall and septum to simulate 20% ischemia. Using the one-compartment model, $S_{f,act}$ in the LV and septal wall was gradually reduced until the LV end-diastolic volume was similar to that of the two-compartment simulation. Then, for both simulations of ischemia and for the baseline simulation, afterload manipulations were performed by increasing and reducing arterial resistance by 20% compared to the reference value, while disabling pressure-flow regulation, thereby constructing an ESPVR. The zero-pressure volume ( $V_{0}$ ) and slope ( $E_{max}$ ) of the ESPVR were calculated.

#### 2.4.2 Regional Myocardial Mechanics: Acute Ischemia and Reperfusion

We hypothesized that acute ischemia and subsequent reperfusion could realistically be simulated by manipulation of the NCVF . To test this hypothesis, we used existing experimental data of regional myocardial deformation measured by sonomicrometry during acute 15-minute left anterior descending (LAD) coronary artery occlusion and subsequent reperfusion in a representative dog (Figure 4A) (Lyseggen et al., 2005). The ultrasonic crystals used for sonomicrometry were implanted in the inner third of the myocardium of the anterior LV wall, which was aligned parallel with the LV long axis. We started with a baseline reference simulation with a similar heart rate (105 bpm) as in the experiment, and we modified MAP to obtain a similar LV peak systolic pressure (84 mmHg). The stroke volume was set to 49 ml to obtain a similar amplitude of segmental shortening as in the experiment. Starting from the baseline simulation, we simulated 15 min of LAD occlusion by increasing the NCVF from 0 to 1 in a myocardial region, which occupied 30% of the total LV wall volume and which was proportionally distributed over the septum and LV free wall. Assuming that the experimental ultrasonic crystals were placed entirely in the anterior LV wall, the strain and segment lengths in the model were calculated within the dysfunctional region located in the LV free wall. The sarcomere length $L_{s}$ in the model was translated to the measured segment length SL by multiplying $L_{s}$ with a constant, calculated from the difference between simulated end-diastolic $L_{s}$ ( $L_{s,ED}$ ) and the experimentally reported end-diastolic segment length ( $SL_{exp,\;ED}$ ) in the baseline reference situation:

$$SL=\;L_{s}.\frac{L_{s,ED}\left(baseline\right)}{SL_{exp,\;ED}\left(baseline\right)}$$ (8)

Parameters $L_{s0,pas}$ and $k_{ECM}$ of $V_{NC}$ (Eq. 3) were tuned to represent the experimental condition after 15 min of ischemia, that is, with similar end-diastolic segment length and systolic stretch amplitude. Starting from the simulation of 15-minute LAD occlusion, we reduced the NCVF from 1 to 0 with steps of 0.2 to simulate contractile recovery after reperfusion.

#### 2.4.3 Regional Myocardial Mechanics: Prolonged Ischemia and Reperfusion

To evaluate whether the two-compartment model could also accurately simulate regional mechanics in ischemic dysfunction with increased stiffness, potentially indicating MI, we attempted to calibrate parameters $L_{s0,pas}$ and $k_{ECM}$ to experimental data of regional myocardial deformation obtained after 4 h of the LAD occlusion followed by reperfusion (Lyseggen et al., 2005). We first calibrated the baseline reference simulation by setting the heart rate to the experimentally measured value of 111 bpm and tuning MAP such that the simulated peak LV systolic pressure was 91 mmHg. The stroke volume was set to 41 ml to obtain a similar amplitude of segmental shortening as in the experiment. Then, we again simulated acute anteroseptal ischemia occupying 30% of the total LV wall volume, with NCVF = 1 and values for $L_{s0,pas}$ and $k_{ECM}$ tuned such that the end-diastolic segment length and systolic stretch amplitude were similar between the model and experiment. Starting from this simulation of acute ischemia, we modified $k_{ECM}$ until the segmental systolic stretch in the model matched the reported stretch after 4 h of ischemia. We further modified $k_{ECM}$ to obtain a similar systolic stretch as measured after 15 min of subsequent reperfusion. In both simulations, $L_{s0,pas}$ was tuned to obtain similar end-diastolic segment lengths between the model and experiment.

### 2.5 Patient-Specific NCVF Estimation

#### 2.5.1 Patient Cohort

Six patients were selected from the DEFI-MI (DEtection of cardiac FIbrosis by LGE magnetic resonance imaging (MRI) and circulating biomarkers in patients with myocardial infarction) cohort. This was a prospective study on first-time MI patients, approved by the local ethics committee (METC: NL45241.041.13) and in accordance with the Declaration of Helsinki. Patients in this study were included after an acute MI, when urgent revascularization was performed. From the measured cohort, we selected patients with narrow QRS (<120 ms), who had a minimum infarct size of 10% of the LV wall mass and a minimum relative LGE percentage of 25% in at least one myocardial AHA segment.

#### 2.5.2 Echocardiographic Measurements

Echocardiography was performed at the baseline (i.e., within 72 h after admission) and at the 6-month follow-up using a commercially available system (Vivid E9, GE Vingmed Ultrasound AS, Horten, Norway). LV volumes and ejection fraction (EF) were acquired by Simpson’s biplane method. Focused loops of the apical four-chamber view, two-chamber view, and three-chamber view were stored for post-processing (GE EchoPAC version 203). Speckle tracking deformation imaging of the LV was performed in 18 segments according to current clinical standards while blinded to the MRI results (Voigt et al., 2015).

#### 2.5.3 Cardiac Magnetic Resonance

At a median of 6 months after primary MI, all patients underwent contrast-enhanced 1.5 Tesla cardiac magnetic resonance (CMR) imaging (Philips Healthcare, Best, Netherlands). LGE image acquisition was performed 15 min after administration of 0.2 ml/kg gadobutrol (Gadovist, Bayer Vital GmbH, Leverkusen, Germany), using prospective ECG-gated sequences of the short axis views from the base to apex, with 5 mm slice thickness. Images were analyzed off-line using Philips ISP9 software (Philips Healthcare, Best, Netherlands). Using the RV insertion points to the interventricular septum as anatomical landmarks, the heart was subdivided into 16 segments according to the model of the American Heart Association (AHA) (Cerqueira et al., 2002), excluding the apical cap. The LGE was quantitatively assessed using the full width at half maximum (FWHM) method, providing a percentage for each of the analyzed segments and the total infarct (scar) size (global %) of the whole LV.

#### 2.5.4 Optimization Algorithm

**FIGURE 2:**
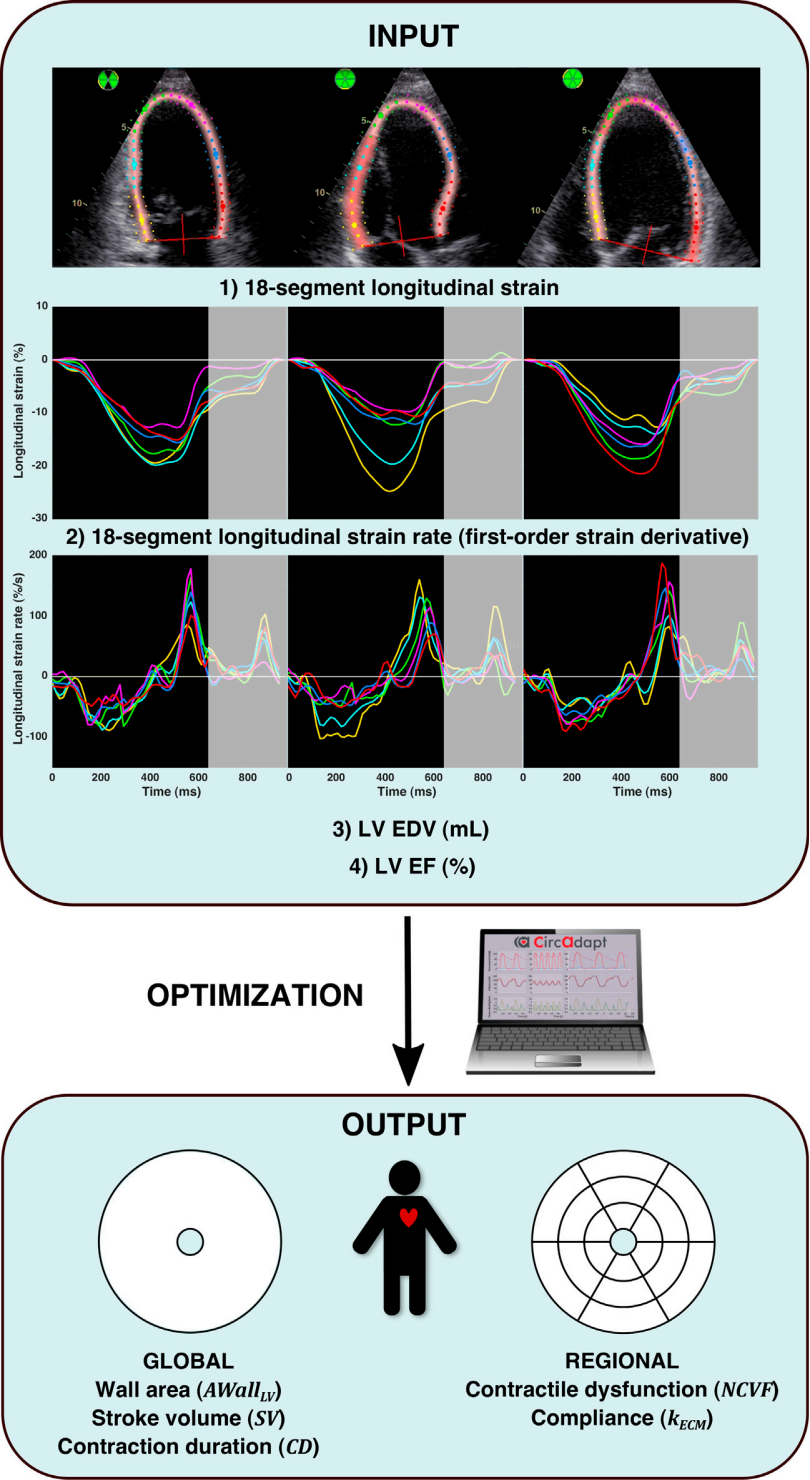
Patient-specific parameter estimation protocol. The input of the optimization algorithm (INPUT) consisted of 1) 18-segment longitudinal strain, 2) 18-segment longitudinal strain rate (calculated as the first-order time derivative of strain), 3) LV end-diastolic volume (EDV), and 4) LV ejection fraction (EF). Strain and strain rate signals were analyzed until early diastole, as indicated by the gray-enhanced parts of the strain and strain rate signals. For numerical optimization (OPTIMIZATION), a multi-swarm particle swarm optimization (MSPSO) algorithm similar to the one previously described in the study by Van Osta et al. (2021) was run, which estimated (OUTPUT): global LV wall area, global stroke volume, global ventricular contraction duration, regional contractile dysfunction, and regional compliance.

To estimate the NCVF on a segmental level, we used a multi-swarm particle swarm optimization (MSPSO) algorithm, as previously described by Van Osta et al. (2021). MSPSO is a stochastic optimization algorithm, which is highly suitable for nonlinear optimization problems. A brief description of the optimization algorithm will be provided here; for further methodological details, we referred to the study by Van Osta et al. (2021). As an input to the optimization algorithm, we used four different echocardiographic measurements (Figure 2, INPUT): 1) the 18-segment longitudinal strain, 2) the 18-segment longitudinal strain rate, calculated as the first-order time-derivative of the strain signal, 3) the LV end-diastolic volume (EDV), and 4) the LV ejection fraction (EF). All four components were normalized in the objective function based on their expected measurement uncertainty, and simulated strains were also scaled to match the global strain amplitude between simulation and measurement. The strain and strain rate signals were analyzed until a pre-defined time point within the diastolic phase, defined as 10% of the cycle time after the moment of 10% global re-lengthening (i.e., global longitudinal strain (GLS) becomes less than 90% of its maximal value). The late diastolic strain was thereby neglected, considering the effects of, for example, drift compensation. Within the time interval analyzed, the sum of squared errors (SSEs) between the model and measurement was calculated, and the SSE was corrected for the number of time points. Parameters estimated in the model (Figure 2, OUTPUT) included the regional NCVF and $k_{ECM}$ , and global LV wall area $A_{wall,LV}$ , global ventricular contraction duration CD , and stroke volume SV . Parameter estimation (Figure 2, OPTIMIZATION) was initiated by performing 1000 quasi-random Monte Carlo (MC) simulations with the heart rate (HR) set to the measured value. For each MC simulation, the objective function was calculated; in addition, for each individual segment, an error was calculated as the sum of errors in the segmental strain and strain rate. A total of 40 initial candidate solutions were selected, which were the best 20 MC simulations based on the objective function and 20 random combinations of the best 20 segmental parameter sets (i.e., NCVF and $k_{ECM}$ ) based on the segmental error. Subswarms were reassigned every 40 iterations, and MSPSO was stopped when either all particles had normalized energy <10^−4^, meaning that within one iteration, all parameters changed by less than 1% of the width of their MC sampling domain, or a maximum number of 1000 iterations was reached.

#### 2.5.5 Model Implementation

The CircAdapt model used in this study has been published before in more detail (Walmsley et al., 2015). To reduce the computational cost, a C++ implementation of this version was used as published before (Van Osta et al., 2020). Equations were linearized using the Newton–Raphson method, and 61 ordinary differential equations were time-integrated using the Adams–Bashford method, with a variable timestep Δt with max (Δ*t*) = 2 ms. MSPSO was performed in MATLAB 2019a (MathWorks, Natick, MA, United States). Simulations ran in parallel on an AMD Ryzen Threadripper 3970X.

## 3 Results

### 3.1 Global Myocardial Mechanics: One-Compartment Vs. Two-Compartment Model

**FIGURE 3:**
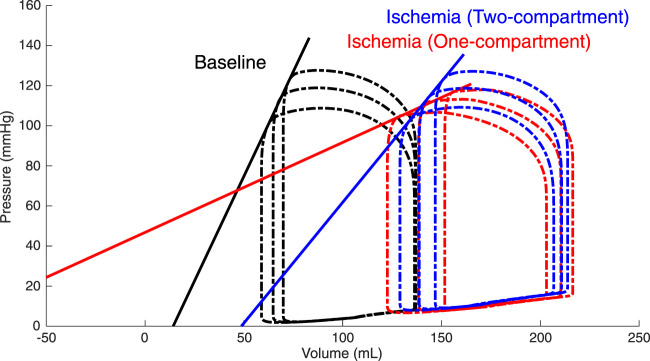
Simulated left ventricular (LV) end-systolic pressure-volume relations (ESPVRs) for a baseline healthy LV (black loops and line) and for an ischemic LV affecting 20% of the LV wall mass modeled by a two-compartment approach (blue loops and line) and a one-compartment approach (red loops and line). ESPVRs were constructed by afterload manipulations through reducing and increasing arterial resistance by 20% compared to the reference resistance. The two-compartment simulation of ischemia demonstrated a rightward shift of the ESPVR with a moderate reduction in the slope, while the one-compartment simulation demonstrated a severe reduction in both the slope and zero-pressure volume.

Constructed ESPVRs for the one- and two-compartment simulations of LV acute regional ischemia are shown in Figure 3. The associated values of $V_{0}$ and $E_{max}$ are provided in Table 1. Compared to the baseline simulation, $E_{max}$ (2.07 mmHg/ml) was moderately reduced for the two-compartment simulation (1.21 mmHg/ml), but it was severely reduced for the one-compartment simulation (0.45 mmHg/ml) of regional ischemia. Furthermore, with respect to the baseline $V_{0}$ (13.2 ml), $V_{0}$ was increased for the two-compartment simulation (46.7 ml), consistent with a rightward shift of the ESPVR, while the one-compartment simulation demonstrated a reduction of $V_{0}$ to −102.4 ml.

**TABLE 1:** Characteristics of the left ventricular (LV) end-systolic pressure–volume relation (ESPVR).

	$V_{0}$ (ml)	$E_{\max}$ (mmHg/ml)
Baseline	13.2	2.07
Ischemia (one-compartment)	−102.4	0.45
Ischemia (two-compartment)	46.7	1.21

### 3.2 Regional Myocardial Mechanics: Acute Ischemia and Reperfusion

**FIGURE 4:**
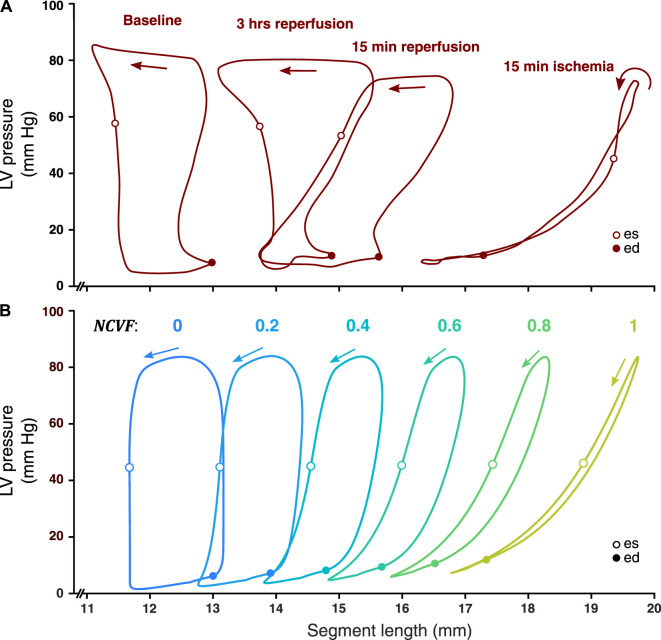
Comparison between measured LV pressure-segment length loops in a representative dog after 15 min of left anterior descending (LAD) coronary artery occlusion, followed by 15 min and 3 h of reperfusion [**(A)**, resketched from Lyseggen et al. (2005)] and simulated LV pressure-segment length loops **(B)** for varying regional contractile dysfunction ( NCVF ). ed, end-diastole; es, end-systole.

**FIGURE 5:**
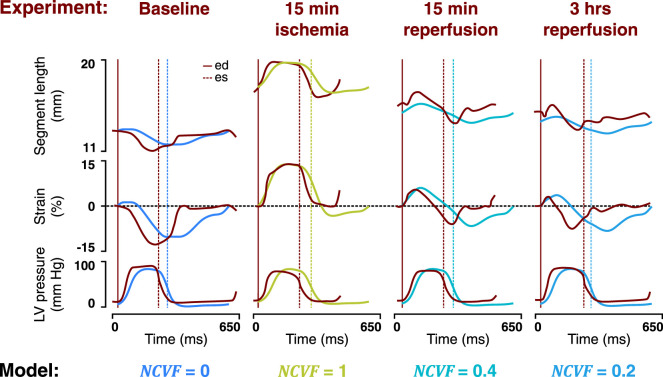
Comparison between the temporal behavior of the measured segment length, strain, and LV pressure in the same representative dog as in Figure 4 after 15 min of left anterior descending (LAD) coronary artery occlusion followed by 15 min and 3 h of reperfusion (dark red tracings, resketched from Lyseggen et al. (2005)) and the simulated segment length, strain, and LV pressure (blue and green tracings) for the best matching severities of regional contractile dysfunction ( NCVF ). ed, end-diastole; es, end-systole.

Simulated LV pressure-segment length loops and corresponding strain patterns at the baseline ( NCVF = 0) and during acute ischemia ( NCVF = 1) were successfully calibrated using the experimental measurements (Figures 4, 5). The calibrated parameter values are provided in Table 2, and an increase in $L_{s0,pas}$ and $k_{ECM}$ during acute ischemia with respect to the baseline is demonstrated. Starting from this simulation of acute ischemia, a reduction of the NCVF shifted the LV pressure-segment length loop leftward, that is, back toward the baseline loop, while the loop area increased. The loops and strain patterns for NCVF values of 0.4 and 0.2 were similar to the measured loops and strain patterns after 15 min and 3 h of reperfusion, respectively (Figures 4, 5).

**TABLE 2:** Calibrated parameter values for the simulations of acute ischemia and reperfusion.

	NCVF (−)	$L_{s0,pas}$ ( μ m)	$k_{ECM}$ (−)
Baseline	0	1.75^a^	10^a^
15-min ischemia	1	2.21	17
15-min reperfusion	0.4	2.21	17
3-h reperfusion	0.2	2.21	17

a Since NCVF=0 , values of $L_{s0,pas}$ and $k_{ECM}$ represent those of the contractile compartment $V_{C}$

### 3.3 Regional Myocardial Mechanics: Prolonged Ischemia and Reperfusion

**FIGURE 6:**
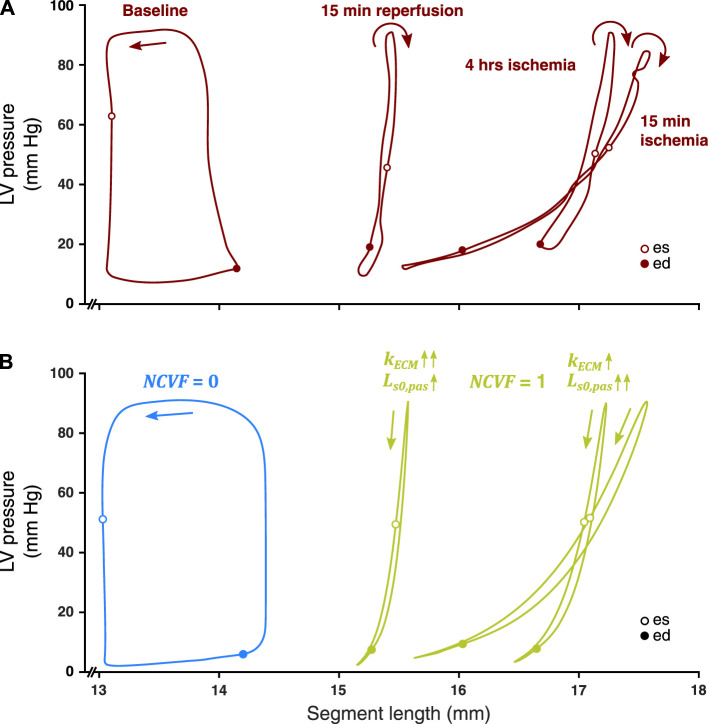
Comparison between measured LV pressure-segment length loops in a representative dog after 15 min and 4 h of left anterior descending (LAD) coronary artery occlusion, followed by 15 min of reperfusion [**(A)**, resketched from Lyseggen et al. (2005)] and simulated LV pressure-segment length loops **(B)** for no contractile dysfunction ( NCVF = 0, blue loop) and complete contractile dysfunction ( NCVF = 1, all three green loops), where nonlinearity parameter ( $k_{ECM}$ ) and zero-stress sarcomere length ( $L_{s0,pas}$ ) were calibrated for the green loops to mimic the experimental measurements (values can be found in Table 3). ed, end-diastole; es, end-systole.

**FIGURE 7:**
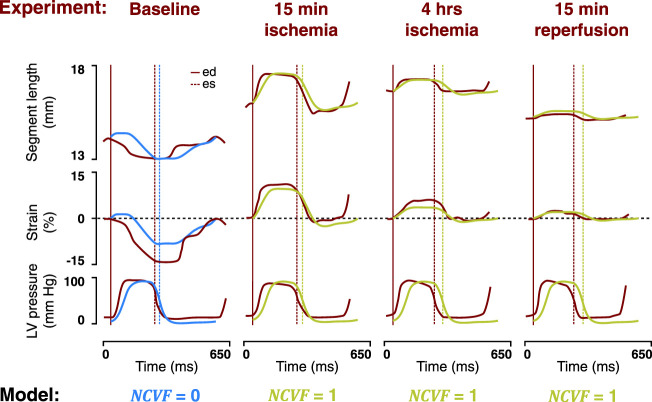
Comparison between the temporal behavior of the measured segment length, strain, and LV pressure in the same representative dog as in Figure 6 after 15 min and 4 h of left anterior descending (LAD) coronary artery occlusion, followed by 15 min of reperfusion (dark red tracings, resketched from Lyseggen et al. (2005)) and the simulated segment length, strain, and LV pressure (blue and green tracings) for no contractile dysfunction ( NCVF = 0) and for three simulations of complete contractile dysfunction ( NCVF = 1), with calibrated values of the nonlinearity parameter ( $k_{ECM}$ ) and zero-stress sarcomere length ( $L_{s0,pas}$ ). ed, end-diastole; es, end-systole.

When starting from the calibrated simulation of acute ischemia, increasing $k_{ECM}$ led to a reduced amplitude of the segmental systolic stretch, while also shifting the LV pressure-segment length loop leftward (Figures 6, 7). Calibration of parameters $L_{s0,pas}$ and $k_{ECM}$ demonstrated that a further increase of both $L_{s0,pas}$ and $k_{ECM}$ was needed to reproduce the measured loop after 4 h of ischemia (Table 3), while a reduction of $L_{s0,pas}$ combined with a further increase of $k_{ECM}$ was needed to reproduce the data after 15 min of subsequent reperfusion.

**TABLE 3:** Calibrated parameter values for the simulations of acute ischemia, prolonged ischemia, and reperfusion.

	NCVF (−)	$L_{s0,pas}$ ( μ m)	$k_{ECM}$ (−)
Baseline	0	1.75^a^	10^a^
15-min ischemia	1	2.01	26
4-h ischemia	1	2.19	70
15-min reperfusion	1	2.03	120

a Since NCVF=0 , values of $L_{s0,pas}$ and $k_{ECM}$ represent those of the contractile compartment $V_{C}$

### 3.4 Patient-specific NCVF Estimation

**FIGURE 8:**
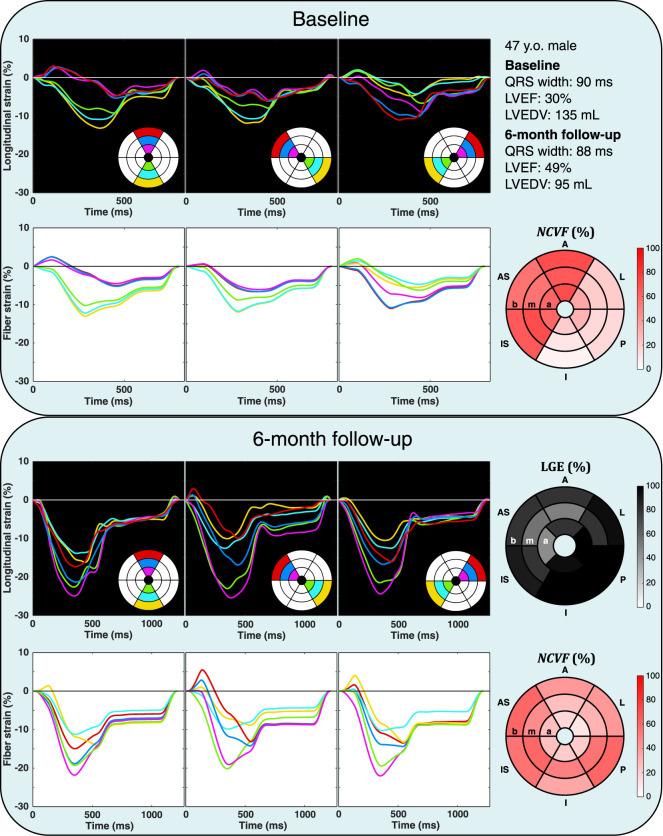
Echocardiographic strain measurements (black panels) and simulated strain patterns obtained using an optimization algorithm (white panels) at the baseline and 6-month follow-up in one patient from the DEFI-MI subcohort (patient 1, a 47-year-old male). Estimated NCVF values at the baseline were higher in the anterior (A), anteroseptal (AS), and inferoseptal (IS) wall segments than in the rest of the heart. After 6 months, the scar tissue was found in the same area using late gadolinium enhancement (LGE). Although contractile dysfunction remained at 6-month follow-up, NCVF values in the A, AS, and IS segments were lower. I, inferior; P, posterior; L, lateral; b, base; m, mid-ventricle; a, apex.

**FIGURE 9:**
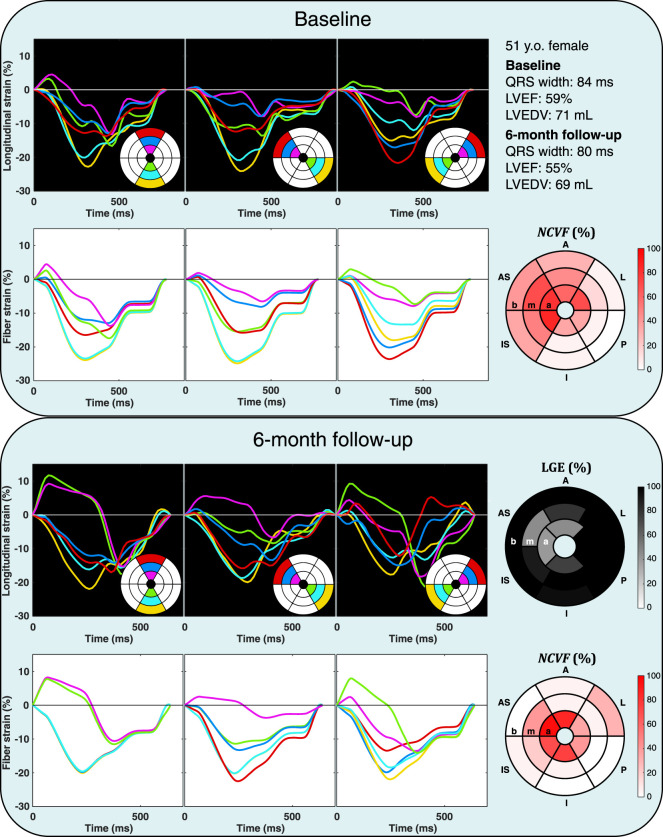
Echocardiographic strain measurements (black panels) and simulated strain patterns obtained using an optimization algorithm (white panels) at the baseline and 6-month follow-up in one patient from the DEFI-MI subcohort (patient 2, a 51-year-old female). Estimated NCVF values at the baseline were higher in the anterior (A), anteroseptal (AS), and inferoseptal (IS) wall segments and in the apical (a) inferior (I), posterior (P), and lateral (L) wall segments than in the rest of the heart. After 6 months, the scar tissue was found using late gadolinium enhancement (LGE) in a part of this dysfunctional area, extending from the apex into the mid-ventricular (m) anteroseptal segment. Increased values of the NCVF were remained in these scarred segments. b, base.

Patient characteristics are summarized in Table 4. Results of the patient-specific estimation of the NCVF for two different patients (patients 1 and 2) are shown in Figures 8, 9, respectively. For all four other patients, results are shown in Supplementary Section S11.3. In both patients 1 and 2, it can be noted that estimated NCVF values were increased in segments with increased systolic stretch or reduced peak systolic strain, both at the baseline and 6-month follow-up. In the acute phase after MI, patient 1 (Figure 8) demonstrated a localized area of increased NCVF in the anterior, anteroseptal, and inferoseptal segments, extending from the base all the way into the apex. At 6-month follow-up, LGE revealed the presence of scar tissue within the same area, especially in the apical septal and mid-ventricular anterior and anteroseptal regions. While increased segmental NCVF values remained after 6 months, the NCVF in the anterior, anteroseptal, and inferoseptal segments were lower than at the baseline. This functional improvement over time was also reflected by an increase of LVEF from 30% at the baseline to 49% at 6-month follow-up. In patient 2 (Figure 9), increased values of NCVF at the baseline were found, especially in the apical anterior and septal segments, but it was extended into the mid-ventricular and basal segments. At 6-month follow-up, while the size of the region with increased NCVF was reduced, severe contractile dysfunction remained in the apex and mid-ventricular anteroseptal region. LGE revealed the presence of a large, transmural scar in this area.

**TABLE 4:** Patient characteristics of the selected DEFI-MI subcohort.

n	6 (83% Male)
Age at baseline (years)	57.6 ± 9.5
Relative infarct size (% of the LV wall mass)	16 ± 6
QRS duration baseline (ms)	90 ± 11
LVEF baseline (%)	49.8 ± 11.5
LVEDV baseline (ml)	100 ± 31
QRS duration 6-month follow-up (ms)	92 ± 13
LVEF 6-month follow-up (%)	50.5 ± 3.5
LVEDV 6-month follow-up (ml)	98 ± 25

**FIGURE 10:**
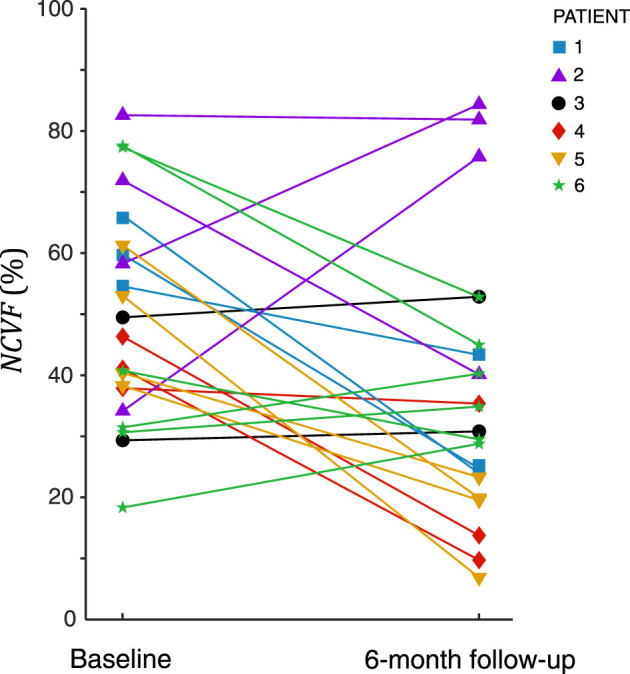
Estimated NCVF values at the baseline and 6-month follow-up in all 22 segments with LGE>25% at 6-month follow-up. Different symbol–color combinations indicate different patients, as shown in the legend. The majority of segments (68%) demonstrated a reduction of the NCVF at 6-month follow-up with respect to the baseline. Results for patient 1 and patient 2 are also shown in Figures 8, 9, respectively. Results for patients 3–6 are shown in Supplementary Section 11.3.

Analysis of all 22 myocardial segments with LGE>25% at 6-month follow-up revealed that in most segments (68%), the NCVF was higher at the baseline than after 6 months (Figure 10). At the baseline, 21 (95%) segments had NCVF >25% and 12 (55%) had NCVF >50%, while at 6-month follow-up, 15 (68%) segments had NCVF >25% and 6 (27%) had NCVF >50%. Average segmental ΔNCVF between the baseline and 6-month follow-up was −16.1 ± 21.8%.

## 4 Discussion

In this computational study, we used the CircAdapt model of the human heart and circulation to test the ability of a lumped two-compartment model to realistically simulate the effects of ischemia-induced contractile dysfunction on ventricular pump and myocardial tissue mechanics. The modeling approach was first evaluated on the level of global LV mechanics by constructing ESPVRs at the baseline and during acute regional ischemia using simulations with a two- and one-compartment modeling approach. Then, simulated regional strains and pressure-length loops for increasing degrees of contractile dysfunction were compared with existing gold standard myocardial deformation measurements in dogs with acute and prolonged ischemia followed by reperfusion. Finally, patient-specific optimizations were performed to estimate the noncontractile volume fraction NCVF in a small cohort of MI patients in the acute and chronic phase after MI, and estimated values were compared with LGE percentages after 6 months. Our results demonstrated that 1) global LV mechanics during regional ischemia were more realistically simulated by the two-compartment than the one-compartment model, 2) manipulation of the NCVF alone could reproduce the experimental deformation data on acute ischemia and subsequent reperfusion, while additional increases of myocardial stiffness were needed to reproduce the deformation data during prolonged ischemia followed by reperfusion, and 3) the patient-specific simulations further supported the idea that this modeling approach can potentially be used for strain-based estimation of regional myocardial contractile dysfunction in patients with cardiovascular disease.

### 4.1 Two-Compartment Model Realistically Simulates Global LV Mechanics

Constructed LV ESPVRs at baseline and during acute regional ischemia using both a one- and two-compartment modeling approach demonstrated that $E_{max}$ was much less affected when a two-compartment model was used (Figure 3; Table 1). This result was expected and is in line with the observations of Sunagawa et al. (1982). In their study, they found no reduction in $E_{max}$ for an ischemic region size of 20%; however, our simulation demonstrated a reduction of $E_{max}$ from 2.07 mmHg/ml to 1.21 mmHg/ml. This lower value of $E_{max}$ in our simulation was likely caused by the relatively compliant passive tissue constitutive behavior, determined by the value of parameter $k_{ECM}$ (Eq. 3). When increasing $k_{ECM}$ , the nonlinearity of the passive stress-sarcomere length relation increases, which causes stiffer passive tissue behavior, thereby increasing $E_{max}$ . This effect of infarct stiffness on the LV systolic function was also previously demonstrated in a modeling study by Fomovsky et al. (2011). It is noted that, at the same time, as a result of the parameterization of the passive tissue behavior, the change in $V_{0}$ with respect to the baseline (+33.5 ml) was more pronounced in our two-compartment model than in the experimental observations, where it was approximately +10 ml for an ischemic region size of 20% (Sunagawa et al., 1982). Qualitatively, however, a similar rightward shift of the ESPVR was observed. The one-compartment simulation of acute regional ischemia demonstrated a severe reduction of $V_{0}$ to a value of −102.4 ml, which is not consistent with experimental observations.

### 4.2 Two-Compartment Model Reproduces Regional Mechanics During Acute Ischemia and Reperfusion

Simulated strain patterns and LV pressure-segment length loops for NCVF values of 1, 0.4, and 0.2 qualitatively agreed with the experimental data on acute ischemia followed by 15 min and 3 h of reperfusion, respectively (Figures 4, 5). While parameters $L_{s0,pas}$ and $k_{ECM}$ were calibrated for the simulation of NCVF = 1 to match the reported end-diastolic segment length and systolic stretch amplitude during acute ischemia, contractile recovery was simulated by reducing NCVF without further modifying $L_{s0,pas}$ and $k_{ECM}$ (Table 2). This result supports NCVF as a parameter quantifying the degree of contractile dysfunction. Calibrated parameter values for the simulation of acute ischemia demonstrated that $L_{s0,pas}$ and $k_{ECM}$ were increased with respect to their baseline values. Increases in $L_{s0,pas}$ during acute ischemia have been reported in previous studies, which have found that ischemic tissue dimensions were larger at matched pressures (Richardson et al., 2015). Adding to the discussion of the previous paragraph, the relatively large increase in $k_{ECM}$ during acute ischemia with respect to the baseline may indicate an inadequate parameterization of $k_{ECM}$ in the baseline simulation, causing the myocardium to be too compliant. While immediate increases in tissue stiffness during acute ischemia have been reported (Theroux et al., 1977), an acute effect of ischemia on tissue passive constitutive behavior has not clearly been established (Holmes et al., 2005). The data showed that after 3 h of reperfusion following acute ischemia, contractile recovery was still incomplete, which was confirmed by our simulations which demonstrated a good agreement with the data for NCVF = 0.2. This persistent dysfunction which follows reperfusion after a short-term period of ischemia has been attributed to myocardial stunning (Braunwald and Kloner, 1982). While it is known that stunning involves complex abnormalities at the cellular level including decreased calcium responsiveness (Gao et al., 1995), which are not explicitly described in our model, the parameter NCVF captured the mechanics during stunning relatively well.

### 4.3 Two-Compartment Model Reproduces Regional Mechanics During Prolonged Ischemia and Reperfusion

Our simulations of prolonged ischemia and subsequent reperfusion demonstrated that $k_{ECM}$ was increased between 15 min and 4 h of ischemia and was even further increased after 15 min of subsequent reperfusion (Figures 6, 7; Table 3). This increased stiffness during prolonged ischemia, and reperfusion is in agreement with measurements of compliance performed in the experiment and was linked to tissue edema as reflected by the increased myocardial water content (Lyseggen et al., 2005). The occurrence of interstitial edema and associated increases of myocardial stiffness during the necrotic phase have also been suggested in other studies (Holmes et al., 2005). At the same time, $L_{s0,pas}$ was increased between our simulations of 15 min and 4 h of ischemia, which may indicate early infarct expansion (Holmes et al., 2005). However, for the simulation of subsequent reperfusion, $L_{s0,pas}$ was again reduced, potentially indicating compaction of the necrotic region during the infarct healing process.

### 4.4 NCVF as a Measure of Ischemia-Induced Contractile Dysfunction

The parameter NCVF is defined in the model as the relative volume of the noncontractile compartment, and increasing NCVF , therefore, leads to increased contractile dysfunction. In the experimental dataset of acute ischemia and subsequent reperfusion, contractile recovery occurred, which was reflected by a reduction of NCVF in the model. However, in the dataset of prolonged ischemia and reperfusion, there was no contractile recovery, and contractile dysfunction remained at the same level ( NCVF = 1). Similarly, the patient-specific NCVF estimations demonstrated that this parameter was increased in segments with systolic stretching or reduced peak systolic strain. Interestingly, in this patient cohort, among all segments with LGE>25% 6 months after MI, NCVF values decreased substantially in most segments from the baseline to 6-month follow-up (Figure 10), suggesting partial recovery of myocardial contractile function over time. A possible explanation for this observed contractile recovery in the majority of segments is that most segments had nontransmural infarction at 6-month follow-up (LGE<50%), and nontransmural infarcts have been associated with functional recovery following revascularization (Ugander et al., 2010). Increased values of NCVF were found especially in segments with LGE>25% after 6 months but were not limited to the LGE-positive segments. It is likely that nonscarred segments with increased NCVF in the acute phase after MI were dysfunctional due to myocardial stunning (Braunwald and Kloner, 1982). Increased NCVF values in nonscarred regions at 6-month follow-up could potentially be related to adverse myocardial remodeling. When we compared our obtained strain data and NCVF estimations with other modeling (Leong et al., 2017) and clinical studies (Zhang et al., 2005; Chan et al., 2006; Becker et al., 2009; Kihlberg et al., 2015; Huttin et al., 2016) of strain in MI, we noticed a common finding that transmural MI causes significant transmural strain abnormalities, including systolic stretching, a reduction of peak systolic strain, and post-systolic shortening. For subendocardial MI, Leong et al. (2017) found in their model that strain was affected in the subendocardial layers, but mid-myocardial and subepicardial strains were preserved. A similar conclusion was reached in a clinical study by Becker et al. (2009), who showed that nontransmural infarction led to greater functional impairment of the endocardial layer than of the epicardial layer. While this transmural heterogeneity in function may exist, multiple studies using strain or strain rate imaging have successfully differentiated subendocardial MI from normal myocardium (Chan et al., 2006; Zhang et al., 2005) and thereby suggest that abnormal strains can be found in segments with subendocardial MI. The same suggestion holds for segments adjacent to an MI region, which have been shown to have a lower peak strain than segments remote from MI (McComb et al., 2015). From these and our own observations, we conclude that the NCVF is a functional parameter, which effectively indicates the degree of ischemia-induced contractile dysfunction; therefore, its use could enhance current diagnostics in patients with cardiovascular diseases. However, at the same time, NCVF alone seems to be unable to differentiate between underlying types of ischemic dysfunction; therefore, it is not suitable to replace LGE. Rather, it could provide useful diagnostic information on its own, or it could be used in addition to LGE.

### 4.5 Limitations

The experimental measurements of Lyseggen et al. (2005) used in our study were acquired by ultrasonic crystals which were aligned parallel to the LV long axis, thereby closely resembling the LV longitudinal strain. However, in our model, we used the one-fiber model to simulate the myofiber strain within a spherical LV geometry; therefore, there can be a systematic discrepancy between simulated and experimental strain values. In the patient-specific optimizations, we corrected for this model discrepancy by normalization of measured and simulated strains to global strain amplitude, but global strain values were not measured in the experiment; hence, no correction was performed. However, the temporal behavior of the experimental and simulated strains was similar; therefore, we expected that this potential mismatch could have had consequences for the quantitative values of calibrated parameters, but the qualitative results remained unaffected. We also assumed that the measurements were obtained from the anterior portion of the LV free wall, implying that we compared these data to simulated strains in the ischemic region located within the LV free wall, thereby disregarding septal deformation. This assumption, if not fully valid, could additionally have caused a minor mismatch in the strain data between the model and experiment. Furthermore, a sensitivity analysis (data not shown) demonstrated that correlations between the NCVF and passive material properties ( $L_{s0,pas}$ and $k_{ECM}$ ) are very small or even absent and that NCVF is the parameter that most significantly determines the contractile function. Since $L_{s0,pas}$ and $k_{ECM}\;$ are parameters which are modified in the noncontractile compartment only, they merely modify the end-diastolic sarcomere length and amplitude of systolic stretch, respectively, of the noncontractile compartment, thereby not impacting intrinsic myocardial contractility. Further research is needed to investigate whether and how $L_{s0,pas}$ and $k_{ECM}\;$ are identifiable from regional strain measurements. The patient data used for this study were retrospectively obtained, and no arterial pressure measurements were available. We assumed that none of the patients had hypertension, and we set pressures in the model to default values. Since strain is known to depend on pre- and afterload (Skulstad et al., 2002; Ferferieva et al., 2012; P. Reant, 2016), this assumption on the patients’ blood pressures could have influenced the strain signals. Furthermore, in our optimization protocol, the number of estimated parameters was limited. We did not estimate, for example, the timing of mechanical activation, which potentially could also differ regionally in patients with MI due to slower electrical conduction in the scar tissue (Richardson et al., 2015). Further research is required to determine whether the set of estimated parameters was sufficient or could potentially be improved. In addition, we only used systolic and early diastolic strains for optimization in this study since we considered the late diastolic strain to be relatively inaccurate due to the effects of, for example, drift compensation. While we believe that the strain interval used is large enough for estimating the relevant parameters in this study, future studies could evaluate whether the late diastolic strain adds relevant information to the objective function.

## 5 Conclusion

We successfully used a two-compartment model for simulation of ventricular pump and myocardial tissue mechanics in ischemic heart disease. Patient-specific optimizations using regional myocardial deformation successfully estimated NCVF in a small cohort of MI patients in the acute and chronic phase after MI, while estimated NCVF values closely approximated the extent of myocardial scar at the follow-up. In future studies, this approach can facilitate deformation imaging–based estimation of myocardial tissue properties in patients with cardiovascular diseases.

## Data Availability

The original contributions presented in the study are included in the article/Supplementary Material, further inquiries can be directed to the corresponding author.
