# Supplementary material for: A Lumped Two-Compartment Model for Simulation of Ventricular Pump and Tissue Mechanics in Ischemic Heart Disease
Source: Front Physiol. 2022 May 11;13:782592. doi: 10.3389/fphys.2022.782592 (PMC9130776; doi:10.3389/fphys.2022.782592)
Supplement: Supplementary file 1 [file DataSheet1.docx]

## Supplementary Figures


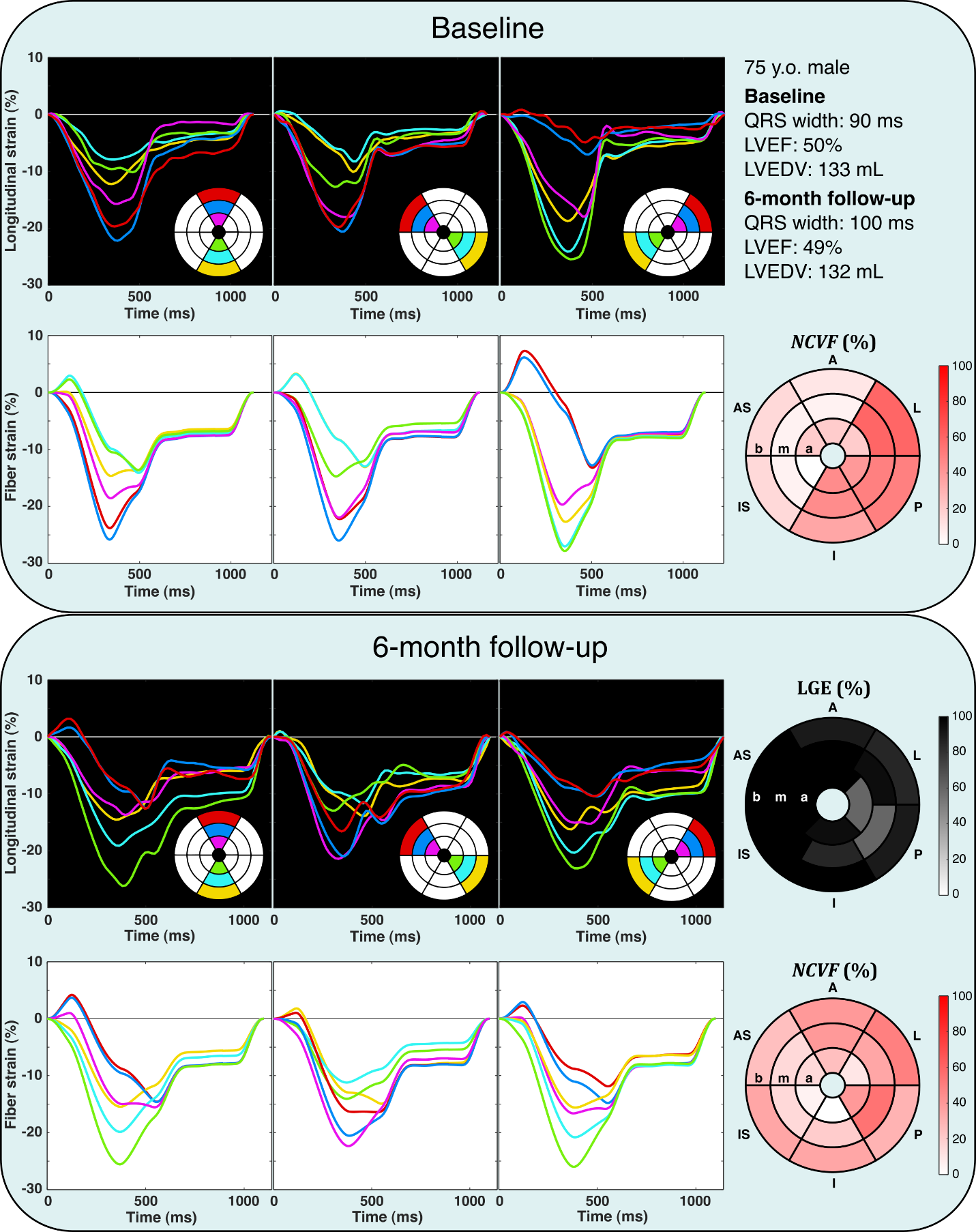


**Supplementary Figure 1**: Echocardiographic strain measurements (black panels), simulated strain patterns obtained using an optimization algorithm (white panels), and corresponding estimated $NCVF$ values (bullseye plots, red color) at baseline and 6-month follow-up, together with late gadolinium enhancement (LGE) at 6-month follow-up (bullseye plot, grey color) in one patient from the DEFI-MI subcohort (PATIENT 3, a 75-year-old male).


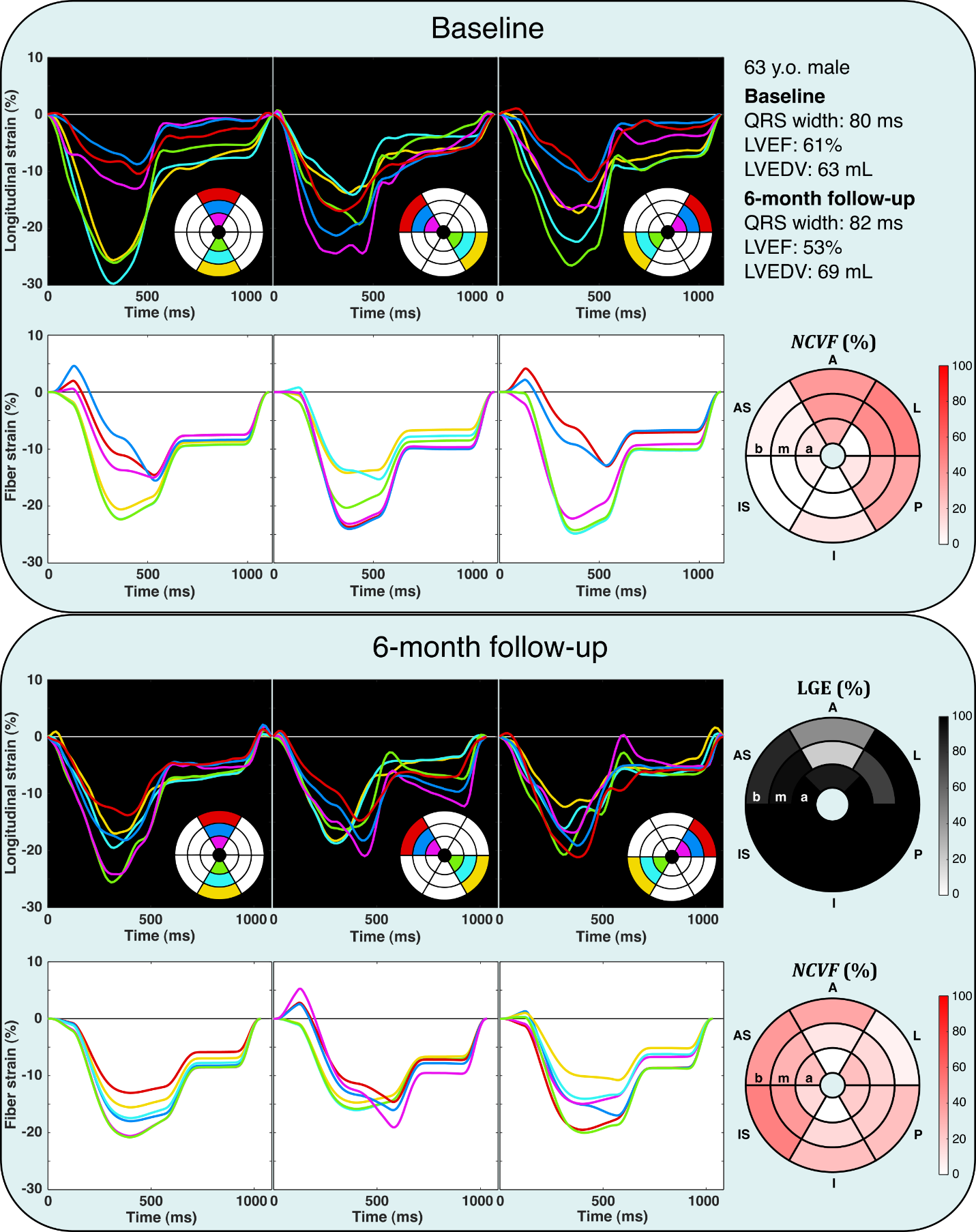


**Supplementary Figure 2**: Echocardiographic strain measurements (black panels), simulated strain patterns obtained using an optimization algorithm (white panels), and corresponding estimated $NCVF$ values (bullseye plots, red color) at baseline and 6-month follow-up, together with late gadolinium enhancement (LGE) at 6-month follow-up (bullseye plot, grey color) in one patient from the DEFI-MI subcohort (PATIENT 4, a 63-year-old male).


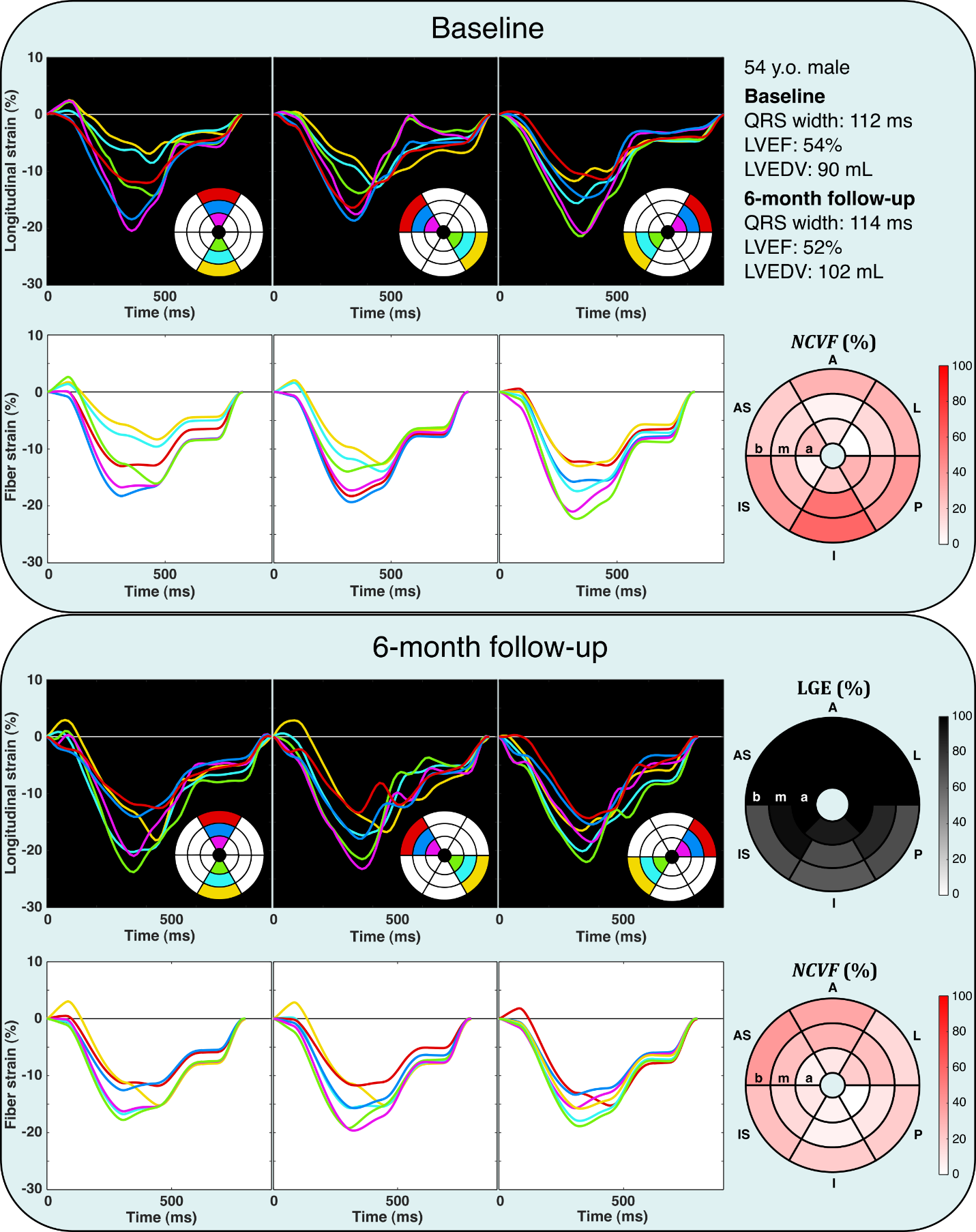


**Supplementary Figure 3**: Echocardiographic strain measurements (black panels), simulated strain patterns obtained using an optimization algorithm (white panels), and corresponding estimated $NCVF$ values (bullseye plots, red color) at baseline and 6-month follow-up, together with late gadolinium enhancement (LGE) at 6-month follow-up (bullseye plot, grey color) in one patient from the DEFI-MI subcohort (PATIENT 5, a 54-year-old male).


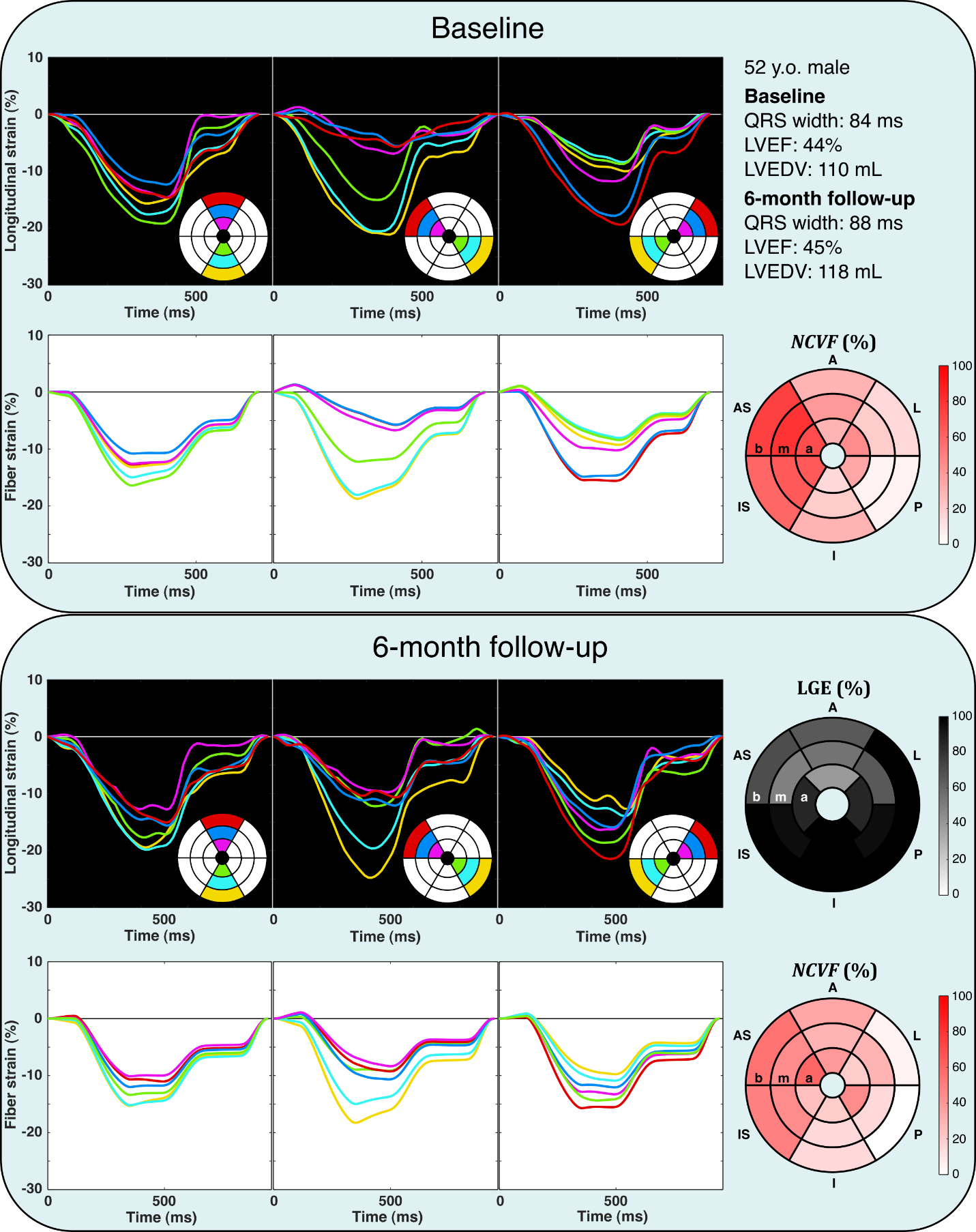


**Supplementary Figure 4**: Echocardiographic strain measurements (black panels), simulated strain patterns obtained using an optimization algorithm (white panels), and corresponding estimated $NCVF$ values (bullseye plots, red color) at baseline and 6-month follow-up, together with late gadolinium enhancement (LGE) at 6-month follow-up (bullseye plot, grey color) in one patient from the DEFI-MI subcohort (PATIENT 6, a 52-year-old male).
